# Concerted control framework for human-exoskeleton co-adaptation using ground reaction forces

**DOI:** 10.1017/wtc.2026.10042

**Published:** 2026-06-02

**Authors:** Vahid Firouzi, Arjang Ahmadi, Dennis Haufe, Andre Seyfarth, Oskar von Stryk, Rolf Findeisen, Maziar Ahmad Sharbafi

**Affiliations:** 1 Lauflabor Locomotion Laboratory, Centre for Cognitive Science, Sport Science Institute, Technical University of Darmstadt, Darmstadt, Germany; 2 Simulation, Systems Optimization and Robotics (SIM) Group, Department of Computer Science,Technical University of Darmstadt, Darmstadt, Germany; 3 Control & Cyber-Physical Systems Laboratory (CCPS), Department of Electrical Engineering and Information Technology,Technical University of Darmstadt, Darmstadt, Germany

**Keywords:** exosuit, control, human-in-the-loop-optimization, human motor control

## Abstract

Effective coordination between the human neuromuscular system and wearable assistive devices remains a key challenge in enhancing gait performance. We propose a concerted control strategy synchronizing biological and artificial actuators using shared feedback. Positioned between centralized (e.g., CPG) and distributed (e.g., reflex-based) control, this approach avoids a central controller by relying on a coordinating signal. Ground reaction force (GRF) emerged as a strong candidate for this role. To implement this concept, we use Force Modulated Compliance (FMC) – a control mechanism that adjusts joint stiffness based on real-time GRF input. FMC has been validated in simulations and robotic platforms, confirming its ability to synchronize joint actuation. We applied this strategy in an active soft biarticular thigh exosuit (BATEX) and tested it in human walking experiments. The GRF-informed controller increased preferred walking speed, advanced the walk-to-run transition, and reduced metabolic cost. These results highlight the effectiveness of GRF-based control in enhancing human-exosuit coordination and aligning assistance with natural gait dynamics. This bioinspired approach offers a scalable framework for real-world locomotion support by harmonizing human and robotic contributions.

## 1. Introduction

Achieving natural and adaptive assistance in human locomotion remains one of the foremost challenges in wearable robotics (Siviy et al., 2023). While recent advances have enabled the design of exoskeletons that reduce metabolic cost or increase walking efficiency (Zhang et al., 2017; Ding et al., 2018), many of these systems rely on control strategies that are locally optimized and phase-dependent, without considering the holistic coordination that underpins biological locomotion. While these methods can reduce metabolic cost or improve walking efficiency in controlled conditions, they often fail to generalize across tasks – such as walking at different speeds, carrying loads, or transitioning to running – and do not fully support human sensorimotor adaptation over time (Poggensee and Collins, 2021; Siviy et al., 2023). In contrast, the human locomotor system achieves robust and efficient movement through a complex interplay of mechanics and control. This system can be broken down into three primary locomotor sub-functions: stance, swing, and balance (Sharbafi et al., 2017). These subsystems do not operate in isolation; rather, they are dynamically synchronized through common sensory inputs and neuromuscular feedback pathways. Drawing from this biological insight, we propose a shift in assistive device control strategy: instead of programming individual components, we seek to orchestrate the entire human-exo system using a central, biologically meaningful signal.

**Figure 1.**
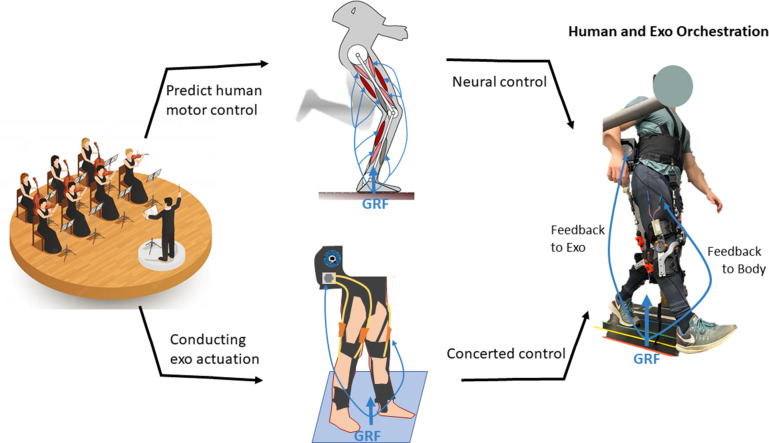
Concerted control concept. The idea is inspired by orchestration through the conductor’s signal. Supported by biomechanical studies, GRF is used in locomotion control (Dietz et al., 1997; Dietz and Duysens, 2000). GRF-based control was implemented for assistive devices (Zhao et al., 2019; Davoodi et al., 2023). The GRF could play the role of the conductor to synchronize human and exo. Figure 1. long description.

We introduce the concept of concerted control – a framework inspired by the idea that human gait operates like an orchestra, with a central “conductor” signal guiding the timing and intensity of various motor outputs (Mohseni et al., 2025). In this context, we hypothesize that the ground reaction force (GRF) serves as the conductor (see Figure 1). GRF is an externally measurable feedback signal that encapsulates key information about load, phase, and intent, making it a powerful candidate for coordinating human and robotic actuation. Research in neurophysiology and biomechanics supports the critical role of load detection in movement coordination (Dietz et al., 1997, 1989; Duysens et al., 2000). The positive force feedback concept also supports the significance of the force signal in the reflex control of muscles, predicting different gaits via neuromuscular models (Geyer et al., 2003). Building on this hypothesis, we define concerted control as a framework in which an assistive device leverages GRF to coordinate its behavior with the user’s natural motor control. This philosophy distinguishes our work from other advanced impedance control strategies, such as learning-based methods (Wang et al., 2024) or online adaptation algorithms (Janna et al., 2024). While those approaches focus on developing sophisticated techniques for how to tune local joint impedance parameters, our concerted control framework addresses the more fundamental question of what coordinating signal should drive the entire system’s behavior. We posit that by using a shared, biologically meaningful signal like GRF, our approach simplifies the control architecture and promotes a more natural human-robot synchrony, which can complement rather than compete with state-of-the-art parameter optimization techniques.

In this article, we present concerted control not just as a technical implementation, but as a bioinspired control philosophy. We implement this framework through a Force Modulated Compliance (FMC) controller, which dynamically adjusts different joints’ impedance (including stiffness and damping) based on GRF feedback. We demonstrate its application across domains – including locomotion modeling, legged robotics, and wearable assistance – to highlight its versatility. Assisted walking with BATEX (BiArticular Thigh EXosuit) (Davoodi et al., 2023; Ahmadi et al., 2025) using concerted control was used to demonstrate the effectiveness of this method to efficiently support human gait speed. Our goal is to position concerted control as a foundational paradigm for synchronizing human and machine in shared movement.

## 2. Materials and methods

In this section, we first introduce the concerted control framework for assistive devices. Then, we introduce the FMC controller as a realization of the concerted control framework. This controller was designed to emulate the biomechanical principles of human motor control by integrating fixed and modulated impedance (spring and damper) and using GRF feedback for dynamic adaptation. Then, we support the hypothesis of GRF-based force modulated compliance (FMC) control by presenting a short survey from three domains: (1) simulation studies showing improved coordination with FMC control in biomechanical models; (2) assistive devices controlled with FMC controllers using GRF feedback; and (3) robotic systems that benefit from FMC controllers. Finally, to investigate our hypothesis, we implemented the FMC control strategy in a soft biarticular thigh exosuit (BATEX).

### 2.1. Embedding concerted control in assistive devices

The goal of this work is to implement and evaluate concerted control as a bioinspired framework for coordinating human-exo interaction. Concerted control is based on the hypothesis that a central sensory signal – namely, the GRF – can synchronize distributed locomotor subfunctions, such as stance support, joint torque production, and swing leg positioning. Unlike conventional phase-based or trajectory-tracking methods, concerted control enables continuous, unified coordination of multiple joints through a single, biologically meaningful feedback signal. Denoting the angle and angular velocity of the joint i , respectively, by $\theta_{i}$ and ${\dot{\theta }}_{i}$ and the ground reaction force by F , the general formulation of the concerted control can be presented by giving individual joint torques $\tau_{i}$ by

(1) $$\tau_{i}=f_{i}\left(\theta_{i},{\dot{\theta }}_{i},F\right).$$

### 2.2. Force modulated compliance (FMC): A control realization of concerted coordination

The FMC controller operationalizes the concerted control framework by regulating joint torques through a combination of constant and modulated compliant components, dynamically scaled by the GRF. This design allows the controller to reflect the mechanical and sensory behavior of human muscles, which adjust stiffness in response to the body load. The FMC control law for the joint torques is given by:

(2) $$\tau_{i}=f_{i}^{s}\left(\theta_{i}\right)+f_{i}^{d}{\dot{\theta }}_{i}+Ff_{i}^{Fs}\left(\theta_{i}\right)+Ff_{i}^{Fd}\left({\dot{\theta }}_{i}\right)$$

in which $f^{s}$ and $f^{Fs}$ represent the general format of (nonlinear) springs by defining their torque-angle functions, while $f^{d}$ and $f^{Fd}$ , define the damping effect using functions of angular velocity. In the most general case, these functions can be defined by nonlinear relations (like the ones in Naseri et al., 2020). However, its simplified version using linear was also used frequently and provided sufficient supporting outcomes in simulations, robots, and eco control:

(3) $$\tau_{i}=k_{i}\left(\theta_{i}^{0}-\theta_{i}\right)+d_{i}{\dot{\theta }}_{i}+c_{i}F\left(\theta_{i}^{0F}-\theta_{i}\right)$$

Here, $k_{i}$ , $c_{i}$ , and $d_{i}$ are, respectively, the fixed linear spring constant, the modulated spring normalized stiffness, and the damper coefficients. Besides, $\theta_{i}^{0}$ and $\theta_{i}^{0F}$ are the rest lengths of the fixed and modulated spring, respectively. The GRF (denoted by F ) is the sensory feedback to coordinate different joints, which should be measured using force sensors (e.g., insole sensors Mohseni et al., 2025). It is important to note that these coefficients are treated as constant gains. The online adaptation is achieved implicitly, as the assistive torque from the modulated component is scaled in real-time by the measured GRF, allowing the joint’s effective compliance to vary dynamically with the user’s or robot’s state. By leveraging GRF in this manner, FMC eliminates the need for explicit gait phase detection or task-specific tuning, making it particularly suited for real-time, adaptive assistance across various locomotion conditions.

### 2.3. Survey on FMC

To evaluate the generality and impact of the FMC controller as a realization of the concerted control framework, we review its implementation and validation across a range of domains. FMC has been applied in biomechanics modeling, robotic locomotion, and assistive devices, where its design aligns with the central hypothesis of concerted control – namely, that a unifying signal such as GRF can orchestrate coordination across joints and subsystems during locomotion.

#### 2.3.1. Biomechanical models

**Figure 2.**
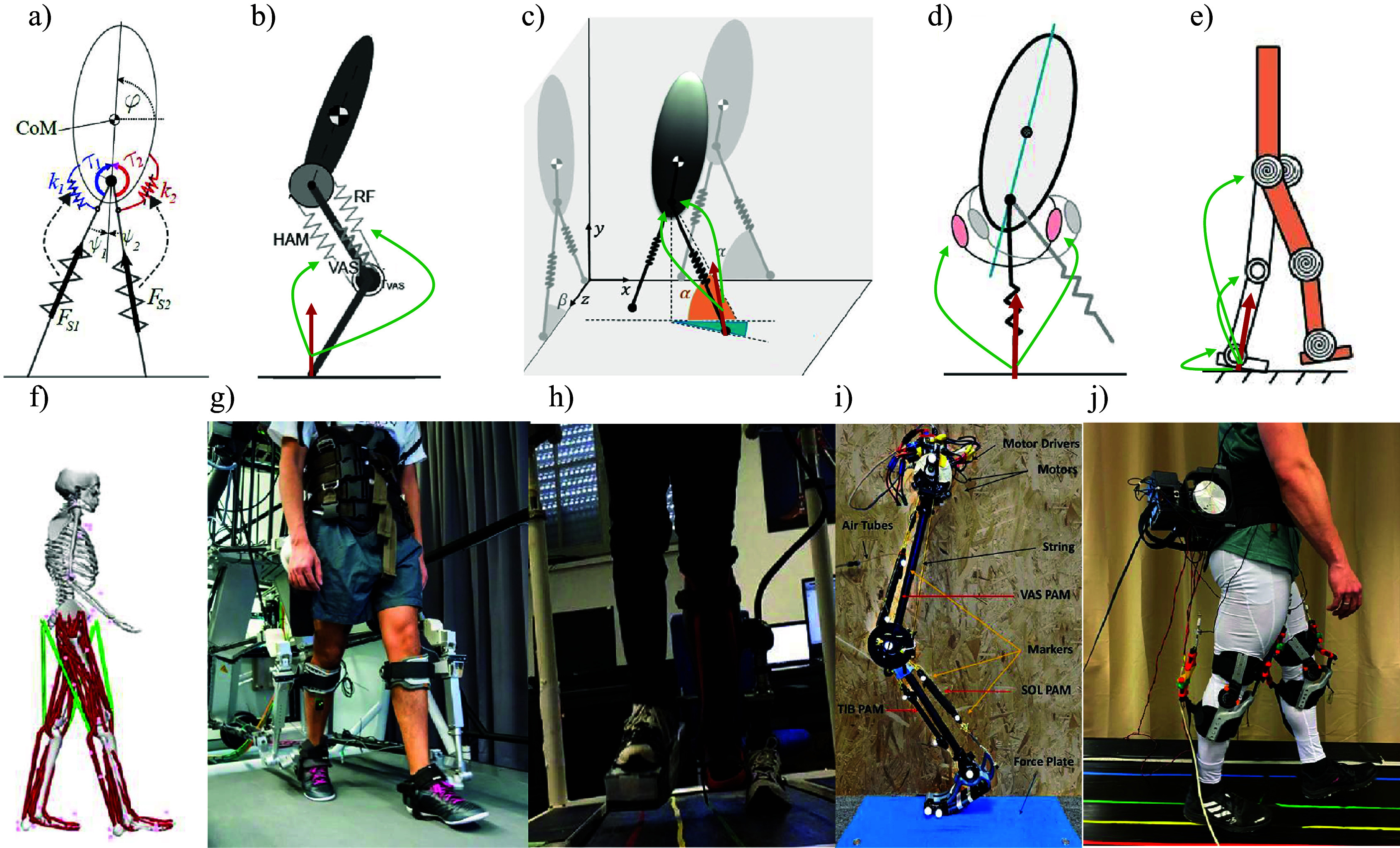
Applications of force-modulated compliant (FMC) control across various domains. The figure illustrates diverse implementations and conceptualizations where the FMC controller is applied. (a) A bipedal spring-loaded inverted pendulum model with trunk (BTSLIP) (Sharbafi and Seyfarth, 2015). (b) A hopping model with segmented leg (Sarmadi et al., 2019). (c) A 3D-BTSLIP gait model (Firouzi et al., 2022; Firouzi et al., 2024a). (d) A BTSLIP model with neuromuscular actuators at the hip (Davoodi et al., 2019). (e) A multi-joint walking model coordinating multiple joints with FMC (Koseki et al., 2025). (f) FMC controller implemented in an experiment-based simulation to actuate a biarticular hip-knee exosuit (Firouzi et al., 2021). (g) FMC controller implemented in the LOPES II exoskeleton (Zhao et al., 2019). (h) Force modulated compliance ankle (FMCA) controller implemented in a powered prosthetic foot (Naseri et al., 2020). (i) Force modulated compliance knee (FMCK) controller implemented in the EPA-hopper robots (Mohseni et al., 2022a; Mohammadi Nejad Rashty et al., 2024). (j) BATEX exosuit (Davoodi et al., 2023) used in this study to validate the concerted control framework. Figure 2. long description.

We have developed various types of gait models, ranging from template-based approaches (Firouzi et al., 2022) (Figure 2a–c) to torque-actuated and neuromuscular-level models (Figure 2d) with segmented legs (Figure 2e) (Davoodi et al., 2019; Koseki et al., 2025). A 3D spring-loaded inverted pendulum (SLIP) model (Shahbazi et al., 2016) was created with a trunk segment (Figure 2c) to represent human walking and running dynamics (Firouzi et al., 2022; 2024a. Hip torques in both sagittal and frontal planes were computed using FMC. In another SLIP-based model, the hip torque generated with Hill-type muscle elements (Figure 2d), and FMC was used to generate muscle activations. Muscle recruitment at the hip was modulated via GRF feedback, demonstrating that FMC could support biologically plausible control (Davoodi et al., 2019). These template-based models were rigorously tested under perturbations to evaluate their stability and robustness (Davoodi et al., 2019; Firouzi et al., 2022).

Recently, we developed a seven-degree-of-freedom (DoF) walking simulation model in MuJoCo (Todorov et al., 2012), consisting of a torso, two hips, knees, and ankles, constrained to move within the sagittal plane (Koseki et al., 2025). In our concerted control framework, during the stance phase, joint stiffness is adjusted according to the GRF based on the FMC control law (Eq. 3). Note that by having zero GRF at the swing leg, the last term in Eq. (3) will be removed, and the controller will be turned into a position control with a PD controller.

#### 2.3.2. Robotic platforms

To evaluate the scalability and generalizability of the controller, it was implemented on a legged robotic platform (Figure 2i) and tested in a hopping experiment. Building upon the FMC controller, the Force Modulated Compliant Knee (FMCK) control method for controlling the knee joint in EPA-Hopper robots was introduced (Mohseni et al. (2022a, 2022b, 2022c). The hopping sequence is divided into flight and stance sub-phases, with foot collision detection occurring between them based on the measured GRF signal. During the stance phase, the constant stiffness and damping in Eq. 3 are set to zero, and the knee joint is controlled using a modulated spring. During the flight phase, the GRF signal is zero, and the knee and hip joints are controlled to predefined target angles using the PD part of Eq. 3, which are manually tuned to ensure the leg reaches the desired posture before ground contact.

**Figure 3.**
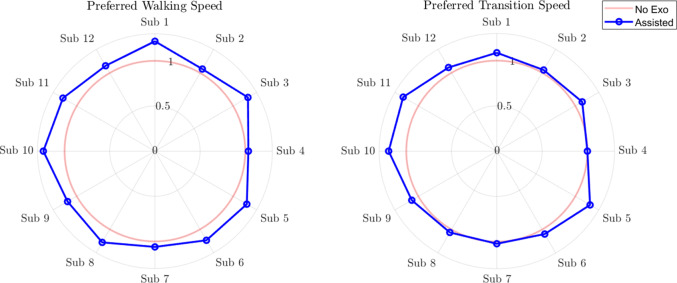
Preferred gait speeds while wearing the exosuit (Assisted) and no exosuit (No Exo) conditions, including the preferred walking speed (PWS) and the preferred walking-to-running transition speed (PTS) for each subject. Figure 3. long description.

#### 2.3.3. Assistive and wearable devices

The FMC controller applied to an exoskeleton (Zhao et al., 2017, 2019) and a powered prosthetic foot (Naseri et al., 2020). Two versions of the FMC controller were implemented in LOPES II (Figure 2g), a robotic exoskeleton capable of applying active hip and knee flexion/extension torques (Meuleman et al., 2015). In one study, FMC was implemented on the hip joint (Zhao et al., 2019). Eight healthy subjects (five females, three males) with no prior exoskeleton experience walked on a treadmill at a fixed speed ( 0.7m/s ) while metabolic costs, muscle activations, and kinematics were measured during the trials. In another study, a force-modulated biarticular muscle was emulated by control of both hip and knee joints (Zhao et al., 2017). In Naseri et al. (2020), a Force-modulated compliant ankle (FMCA) control strategy is used to regulate a powered prosthetic foot’s torque during walking. FMCA approximates the required ankle torque based on vertical GRF, ankle angles, and angular velocities. The same driven equation was used to approximate the average ankle torque of 21 participants (data from Lipfert, 2010) for walking at different speeds 50, 75, and 100% of PTS Preferred walking to running Transition Speed). This FMCA controller was then implemented on a powered prosthetic foot (Ruggedized Odyssey Ankle, Figure 2h) and tested with a non-amputee subject walking using a bipassed system for the human ankle joint on a treadmill at preferred walking speed (PWS, 1.3 m/s about 75% PTS).

### 2.4. FMC controller implemented in BATEX

The BATEX Exosuit (Figure 2j) includes one motor per leg that actuates both hip and knee joints using a series elastic actuator (SEA). This design mimics the function of the thigh biarticular muscles in the human leg (rectus femoris and hamstrings). In Firouzi et al. (2021), we applied FMC to control the actuation of BATEX in simulation. Inspired by our previous studies (Sharbafi et al., 2017, locking the motor in the swing phase alters the SEA to spring, which is sufficient to apply the required hip and knee torques of the concerted control equation. In Davoodi et al. (2023), we have implemented the abovementioned controller on the BATEX exosuit.

We have conducted assisted walking experiments ( n=12 ; 4 female, 8 male; age, 25±4years ; mass, 77.2±12kg ; height, 1.83±0.06m ; mean ± SD) to investigate how the proposed control framework could support human walking. In the first round, we identified the preferred walking speed (PWS) and the preferred transition speed (PTS) from walking to running for each subject. In the second step, we have applied the FMC controller (see Davoodi et al., 2023 for details) on the exosuit, and the users were able to adjust the control gain at each specific speed using a joystick. In the third step, we identified the PWS and PTS for assisted walking. Finally, we measured metabolic rate, kinematic, and GRFs during 8 min of assisted walking at the unassisted PWS.

To compare PWS and PTS between the assisted and no-exosuit conditions, we performed paired sample *t*-tests on within-subject differences (*n* = 12). For metabolic cost, which was assessed under three conditions (No Exo, Zero Torque, and Assisted), we conducted a repeated-measures ANOVA to evaluate differences across conditions.

## 3. Results

In this section, we begin with a brief overview of the results related to the applications of the FMC controller across various domains. Following this, we explore the results of a newly developed model based on the concerted control concept and the experimental results of BATEX for gait assistance.

### 3.1. Applications of the FMC controller

The overview of FMC applications in Table 1 demonstrates that FMC has been successfully applied in both experimental studies and simulation environments. Its use across various gaits (e.g., walking, running, and hopping) highlights FMC’s potential for diverse locomotion tasks. Furthermore, FMC has been employed to control different degrees of freedom (DoFs), showcasing the effectiveness of GRF-based concerted control in synchronizing multiple joints.

**Table 1.** Overview of the applications of the force-modulated compliant (FMC) controller across various domains Table 1. long description.

App.	Ref.	Sim vs Exp	Gait	Joint(s)	Main outcome
**Model**	Sarmadi et al. (2019)	Sim	Hopping	Hip Flx/Ext, Knee	Simplified motor control and enhanced robustness
	Sharbafi and Seyfarth (2015)	Sim	Walking	Hip Flx/Ext	Stable and robust walking
	Firouzi et al. (2024a)	Sim	Walking	Hip Flx/Ext, Hip Abd/Add	3D walking gait is generated
	Davoodi et al. (2019)	Sim	Walking	Hip Flx/Ext	Human-like, robust walking
	Koseki et al. (2025)	Sim	Walking	Hip Flx/Ext, Knee, Ankle	Robust, human-like walking gaits at various speeds with Concerted control
	Firouzi et al. (2022)	Sim	Running	Hip Flx/Ext, Hip Abd/Add	Human-like running and perturbation rejection
**Assistive devices**	Zhao et al. (2019)	Exp	Walking	Hip Flx/Ext	Reduced muscle activity
	Zhao et al. (2017)	Exp	Walking	Hip Flx/Ext, Knee	Reduced muscle activity and metabolic cost
	Naseri et al. (2020)	Exp	Walking	Ankle	Human-like ankle torques and stable walking
	Firouzi et al. (2021)	Sim	Walking	Hip Flx/Ext, Knee	Reduced metabolic cost and adaptation to various tasks
	Firouzi et al. (2022)	Sim	Walking	Hip Abd/Add	Reduced metabolic cost and adaptability to various tasks
**Robot**	Mohseni et al. (2022c)	Sim/Exp	Hopping	Knee	Increased the robot’s operational efficiency.
	Mohammadi Nejad Rashty et al. (2024)	Exp	Hopping	Knee	Mimicking human hopping with FMCK

*Note:* The table categorizes the studies into three main applications: developing gait models, assistive devices, and robot design. For each study, the type of evaluation (simulation or experiment), the gait type investigated, the joint(s) analyzed, and the main outcome are specified.

### 3.2. Assistive device

The implementation of the FMC controller in the BATEX provides direct experimental support for the concerted control framework. By using GRF as the coordinating signal, the system achieved synchronized joint actuation across the hip and knee without relying on explicit gait phase detection or joint-specific control trajectories. This enabled a natural coupling between human motion and exosuit assistance, with assistance profiles emerging adaptively in response to user-generated GRF patterns.

During walking trials, participants demonstrated enhanced locomotor performance with the exosuit operating under concerted control. Figure 3 demonstrates increases in preferred walking speed (PWS) for all subjects. The BATEX with concerted control also increased the preferred transition speed (PTS) from walking to running for all subjects, except two (sub4 and sub7). On average, the PWS and PTS were increased by 14.3% and 9%, respectively (see Supplementary Material for raw data). Both increments were statistically significant. These shifts suggest that users could walk more comfortably and confidently at higher speeds. It is likely due to improved timing and magnitude of assistance that aligned with their natural gait dynamics.

**Figure 4.**
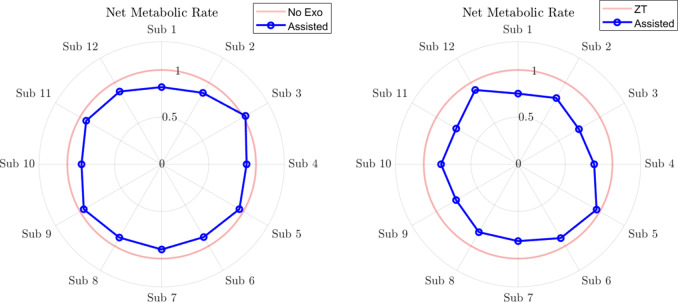
Metabolic cost measured at the PWS was evaluated for three conditions: no exosuit (No Exo), zero-torque exosuit (ZT), and active exosuit assistance (Assisted) for each subject. Figure 4. long description.

In addition to enabling users to walk faster, this coordinated assistance provided significant metabolic benefits at the user’s preferred walking speed. Figure 4 illustrates the ratio of metabolic cost in the assisted case with concerted control to two unassisted cases, normal walking (No Exo) and with exo exerting zero torque (ZT). As can be seen, this ratio is <1 for almost all the subjects in both cases, meaning reduced metabolic costs in the assisted case. On average, the exosuit with concerted control reduced metabolic cost by 17.9% compared to the zero torque (ZT) condition. The net improvement relative to normal walking was a 9.5% reduction. Both reductions are statistically significant. These metabolic savings were achieved without predefined actuation profiles, reinforcing the principle that GRF can drive effective, system-wide coordination through real-time feedback rather than open-loop optimization.

## 4. Discussion

Biological systems control locomotion using feedback (force/displacement/speed) and feedforward motor patterns generated by neural networks (Geyer and Herr, 2010). This combination, through the muscle-tendon-complex (MTC), is known as sensor-motor mapping (Schumacher and Seyfarth, 2017). In contrast to this distributed motor control architecture, centralized control is achieved through spinal central pattern generators (CPGs) (Ijspeert, 2008), which produce rhythmic activation patterns. CPGs coordinate different body segments, while distributed reflex pathways fine-tune motor output based on local sensory feedback (Ijspeert and Daley, 2023). In this study, concerted control was introduced as an alternative approach for coordinating different players of human motor control, which can also sync them with assistive systems. We presented evidence for the applicability of the leg force for motor control in locomotion and also preliminary steps of its implementation for gait assistance. The simulation results of generating human-like patterns with a simple controller supported the concept of implicit coordination of different locomotor subfunctions through a conducting signal (GRF) (Sarmadi et al., 2019; Koseki et al., 2025; Mohseni, 2025). Assisted walking with the BATEX exosuit also confirmed the usefulness of this method in orchestrating human and an assistive device. Simultaneous improvement in agility (e.g., walking speed) and efficiency (metabolic rate) is an outcome of our bioinspired controller, which has been achieved through matching human and exo motor control following the concerted control idea. In the following, we discuss the potential advantages of concerted control in designing assistive devices.

### 4.1. Improved human-robot interaction

Utilizing GRF as sensory feedback allows the exoskeleton to react to the user’s movements in a bio-inspired manner, promoting a more collaborative interaction instead of a master–slave relationship. By synchronizing with the user’s actions, the exoskeleton could complement the natural gait cycle, potentially enhancing user comfort and overall performance.

### 4.2. Adaptability

Employing GRF feedback in exoskeleton control facilitates adaptation to new conditions, including varying speeds, load carriage, and environmental changes. As demonstrated in Firouzi et al. (2021), Firouzi et al. (2022) between the optimized FMC controller and model-free optimal controller for normal walking, FMC outperformed the model-free optimal controller when the gait changed to a load-carrying task. This adaptability to new conditions is due to the controller’s ability to use GRF as an informative sensory signal. The inherent variability of GRF patterns during different tasks provides valuable information about the user’s current state, allowing the exoskeleton to adjust its assistance level accordingly. This adaptability is crucial for real-world applications, ensuring the exoskeleton remains effective in different scenarios.

### 4.3. Reducing effort

FMC control has been shown to reduce metabolic costs during walking. This reduction can be attributed to the controller’s ability to provide assistance that minimizes the user’s muscular effort. Studies involving the FMC controller have demonstrated significant reductions in muscle activation and metabolic costs (Zhao et al., 2019, 2017) at different conditions (e.g., normal walking and load carrying), suggesting that GRF feedback allows the exoskeleton to effectively offload some of the user’s workload and adapt to different conditions (Firouzi et al., 2021; 2022). Our experimental results demonstrated reduced walking effort at the PWS. Previous studies have shown that humans naturally select a walking speed that minimizes the cost of transport (Srinivasan, 2009). Given this fact and the observed increase in PWS with our assistive system employing concerted control, we predict that it may be possible to increase walking speed while simultaneously reducing the cost of transport (metabolic cost per unit distance). This potential benefit warrants further investigation in future studies.

### 4.4. Device energy efficiency

Compared to active constant compliance control, GRF-modulated compliance leads to smoother motor torques and reduced peak power requirements for the exoskeleton, indicating higher efficiency (Zhao et al., 2019). By adapting both GRF-modulated compliance and passive elements, the assistive device can optimize its energy expenditure and minimize unnecessary actuation, contributing to extended battery life and improved overall performance (Firouzi et al., 2021).

### 4.5. Simplifying control complexity

A significant advantage of the FMC controller lies in its ability to simplify control complexity by eliminating the need for precise gait phase detection. The GRF signal provides inherent information about the user’s gait cycle, enabling the exoskeleton to adapt its assistance without relying on external sensors or complex algorithms for phase identification. This simplification can reduce the computational burden and improve the real-time responsiveness of the exoskeleton. Furthermore, in our experiments, tuning only one control parameter (modulated stiffness $c_{0}$ ) by the user suffices to adapt to different speeds. This minimal parameter adaptation can potentially elevate the adaptability of assistive systems for real-world applications.

### 4.6. Potential for personalized assistance

GRF feedback allows for personalized assistance by accounting for individual variations in gait patterns. This personalized approach can enhance the effectiveness of the exoskeleton, catering to the specific needs and capabilities of each user. The aforementioned controller simplification and reducing adaptable control parameters could facilitate personalization. Moreover, concerted control with a minimal set of adjustable parameters may improve the efficiency and quality of human-in-the-loop optimization (Zhang et al., 2017).

## 5. Conclusion and outlook

The concept of concerted control, leveraging GRF as a unifying signal, offers a promising pathway for advancing wearable assistance technology. By utilizing GRFs to synchronize actuation at multiple joints, such as the hip, knee, and ankle, exoskeletons could more effectively integrate with the user’s natural biomechanics. This biomimetic approach provides a foundation for achieving seamless human-exo co-adaptation, addressing critical challenges in mobility assistance and rehabilitation.

The presented approach is a first step requiring further investigation. The interplay between central and peripheral nervous systems in locomotion control highlights the complementary roles of central pattern generators (CPGs) and reflex-based mechanisms (Thandiackal et al., 2021; Di Russo et al., 2023). Reflex-based control, even without predefined feedforward circuits, has demonstrated the ability to generate robust gaits (Song and Geyer, 2015; Firouzi et al., 2024b. However, combining central mechanisms with peripheral feedback, such as continuous GRF sensing, has been shown to create more robust and adaptive locomotion systems (Thandiackal et al., 2021). For instance, incorporating GRF feedback into CPGs enables self-organized locomotion, a principle that is underutilized in current assistive device control strategies (Sun et al., 2021).

Future research should investigate the adaptability of GRF-based concerted control in dynamically tailoring itself to individual users and diverse locomotion tasks, such as walking, running (Mohseni et al., 2025), and stair climbing. This approach presents exciting possibilities for developing multi-joint exoskeletons capable of actively coordinating joint movements, emulating the natural harmony of the human musculoskeletal system (Firouzi et al., 2025). This approach also has the potential to coordinate different joints in legged robots, which we are investigating in future work.

## Supporting information

10.1017/wtc.2026.10042.sm001 Firouzi et al. supplementary material Firouzi et al. supplementary material

## Data Availability

The authors confirm that the data supporting the findings of this study are available within the article and its Supplementary Materials.

## Supplementary material

The supplementary material for this article can be found at http://doi.org/10.1017/wtc.2026.10042.

## Figure 1. Long description

A conceptual flowchart detailing the “Human and Exo Orchestration” architecture, illustrated using a functional analogy to an orchestral conductor. On the far left, an illustration depicts a musical orchestra on a circular wooden stage with a conductor raising a baton. Two black arrows branch out to the right from this conductor illustration: the upper arrow is labeled “Predict human motor control” and points to a grey biomechanical leg model showing activated leg muscle groups (labeled HAM, VAS, and RF) experiencing an upward vertical Ground Reaction Force (GRF) arrow at the foot contact point. The lower arrow from the orchestra is labeled “Conducting exo actuation” and points to a schematic diagram of a lightweight lower-limb exoskeleton frame attached to a pair of legs, also showing an upward vertical GRF arrow at the base. These two intermediate schematic paths converge on the far right into a comprehensive illustration labeled “Human and Exo Orchestration”. This image displays a side profile photo of a human participant walking while wearing a soft biarticular thigh exosuit (BATEX) on a specialized treadmill platform. Blue curved feedback loops outline the control synchronization: a “Neural control” arrow feeds downwards into the human, “Feedback to Exo” maps the vertical GRF from the sensor plate back into the exosuit processing unit, and “Feedback to Body” routes information back to the user’s sensorimotor pathways. At the base of the user’s shoe, a detailed blue three-axis coordinate arrow system identifies the real-time localized Ground Reaction Force (GRF) serving as the unifying coordinating signal.

Navigate back to Figure 1..

## Figure 2. Long description

A composite ten-panel grid layout consisting of two rows labeled sequentially from a through j, demonstrating various computer models and physical experimental platforms implementing the FMC controller. Row 1 (a to e) - Biomechanical and Template Models:a) A 2D schematic diagram of a bipedal spring-loaded inverted pendulum model with an elliptical trunk (BTSLIP), detailing the center of mass (CoM), trunk tilt angle (φ), hip spring constants (k_1_, k_2_), and linear leg forces (F_leg1_, F_leg2_). b) A segmented mechanical hopping leg model consisting of a main upper torso weight and a three-segment leg utilizing rotational springs labeled for the Hamstrings (HAM), Vastus (VAS), and Rectus Femoris (RF) muscular equivalents, with a vertical GRF force arrow acting on the toe contact point. c) A 3D-BTSLIP gait template model with projected leg shadows on orthogonal planes to indicate multi-planar walking. d) A Template model actuating hip joint with muscles combined with a linear leg spring. e) A multi-joint sagittal walking model displaying a multi-segmented trunk, thigh, shank, and foot assembly, with green directional loops highlighting multi-joint coordination driven by ground reaction force feedback. Row 2 (f to j) - Simulations, Exoskeletons, Robots, and Prosthetics: f) A side-view biomechanical simulation rendering of a human skeleton with muscle groups highlighted in bright red overlayed with green lines representing a simulated biarticular hip-knee exosuit. g) A photographic front-angle view of a human subject walking inside the LOPES II rigid robotic exoskeleton frame on a laboratory treadmill. h) A close-up photograph focusing on the lower legs of a user walking on a treadmill, wearing a specialized powered prosthetic foot assembly (FMCA) on the right foot. i) A photograph of the physical EPA-hopper robotic leg standing on a blue force sensor. j) A side profile photograph of a human subject walking inside a laboratory setting, wearing the soft BiArticular Thigh Exosuit (BATEX) configuration used for validation in this study, featuring a waist-mounted motor driver housing and routing lines down the thigh to the knee.

Navigate back to Figure 2..

## Figure 3. Long description

The left radar chart is titled Preferred Walking Speed. The chart has twelve axes labeled clockwise from the top as Sub 1 through Sub 12. The radial axis is marked at 0, 0.5, and 1. Two data series are shown: a thin red line labeled No Exo and a thick blue line with circular markers labeled Assisted. For all subjects, the blue Assisted line is outside the red No Exo line, indicating higher preferred walking speeds with assistance. The right radar chart is titled Preferred Transition Speed, with the same subject and radial axis arrangement. Again, the blue Assisted line is consistently outside the red No Exo line for all subjects, showing higher preferred transition speeds with the exosuit. The legend in the upper right of the right panel identifies the line styles for No Exo and Assisted.

Navigate back to Figure 3..

## Table 1. Long description

The table is organized into six columns: Application, Reference, Simulation versus Experiment, Gait, Joint or Joints, and Main outcome. The first group, Model, includes six studies: Sarmadi et al. 2019 (Sim, Hopping, Hip Flexion/Extension and Knee, Simplified motor control and enhanced robustness); Sharbafi and Seyfarth 2015 (Sim, Walking, Hip Flexion/Extension, Stable and robust walking); Firouzi et al. 2024a (Sim, Walking, Hip Flexion/Extension and Hip Abduction/Adduction, 3D walking gait is generated); Davoodi et al. 2019 (Sim, Walking, Hip Flexion/Extension, Human-like, robust walking); Koseki et al. 2025 (Sim, Walking, Hip Flexion/Extension, Knee, Ankle, Robust, human-like walking gaits at various speeds with concerted control); Firouzi et al. 2022 (Sim, Running, Hip Flexion/Extension and Hip Abduction/Adduction, Human-like running and perturbation rejection). The second group, Assistive devices, includes five studies: Zhao et al. 2019 (Exp, Walking, Hip Flexion/Extension, Reduced muscle activity); Zhao et al. 2017 (Exp, Walking, Hip Flexion/Extension and Knee, Reduced muscle activity and metabolic cost); Naseri et al. 2020 (Exp, Walking, Ankle, Human-like ankle torques and stable walking); Firouzi et al. 2021 (Sim, Walking, Hip Flexion/Extension and Knee, Reduced metabolic cost and adaptation to various tasks); Firouzi et al. 2022 (Sim, Walking, Hip Abduction/Adduction, Reduced metabolic cost and adaptability to various tasks). The third group, Robot, includes two studies: Mohseni et al. 2022 (Sim/Exp, Hopping, Knee, Increased the robot’s operational efficiency); Mohammadi Nejad Rashty et al. 2024 (Exp, Hopping, Knee, Mimicking human hopping with FMCK). The table footnote explains that studies are categorized by application, with evaluation type, gait, joints, and main outcome specified.

Navigate back to Table 1..

## Figure 4. Long description

The left panel is a radar chart labeled Net Metabolic Rate, with axes for Sub 1 through Sub 12 arranged clockwise. Concentric rings are labeled 0, 0.5, and 1. The red line represents No Exo, forming a circular shape at the 1 ring. The blue line with circles represents Assisted, consistently inside the red line, indicating lower net metabolic rates for all subjects. The legend at the top right identifies No Exo in red and Assisted in blue. The right panel is similarly structured, with the red line labeled ZT for zero-torque exosuit and the blue line for Assisted. Again, the Assisted line is inside the ZT line for all subjects, showing reduced net metabolic rates. The legend at the top right identifies ZT in red and Assisted in blue. In both panels, the reduction in net metabolic rate with Assisted is consistent across all twelve subjects.

Navigate back to Figure 4..
